# Escher-FBA: a web application for interactive flux balance analysis

**DOI:** 10.1186/s12918-018-0607-5

**Published:** 2018-09-26

**Authors:** Elliot Rowe, Bernhard O. Palsson, Zachary A. King

**Affiliations:** 1 Department of Bioengineering, University of California, San Diego, La Jolla, USA; 2 Bioinformatics and Systems Biology Program, University of California, San Diego, La Jolla, USA; 3 Department of Pediatrics, University of California, San Diego, La Jolla, CA USA; 4 Novo Nordisk Foundation Center for Biosustainability, Technical University of Denmark, Kemitorvet, Building 220, 2800 Kongens, Lyngby, Denmark

**Keywords:** Constraint-based modeling, Flux balance analysis, Escher, Visualization, Metabolism, Web application

## Abstract

**Background:**

Flux balance analysis (FBA) is a widely-used method for analyzing metabolic networks. However, most existing tools that implement FBA require downloading software and writing code. Furthermore, FBA generates predictions for metabolic networks with thousands of components, so meaningful changes in FBA solutions can be difficult to identify. These challenges make it difficult for beginners to learn how FBA works.

**Results:**

To meet this need, we present Escher-FBA, a web application for interactive FBA simulations within a pathway visualization. Escher-FBA allows users to set flux bounds, knock out reactions, change objective functions, upload metabolic models, and generate high-quality figures without downloading software or writing code. We provide detailed instructions on how to use Escher-FBA to replicate several FBA simulations that generate real scientific hypotheses.

**Conclusions:**

We designed Escher-FBA to be as intuitive as possible so that users can quickly and easily understand the core concepts of FBA. The web application can be accessed at https://sbrg.github.io/escher-fba.

**Electronic supplementary material:**

The online version of this article (10.1186/s12918-018-0607-5) contains supplementary material, which is available to authorized users.

## Background

The constraint-based modeling approach to studying metabolic networks has led to a great variety of applications, from understanding metabolic gene essentiality, stress tolerance, and gene regulation to designing microbial cell factory [1]. The simplest and most popular constraint-based method is flux balance analysis (FBA) [2], and many derivative approaches draw their value from extending the elegant insights of FBA [3]. Most tools for FBA simulation require software downloads, have significant learning curves, and require computer programming. However, FBA has broad interest, so there is a great value in simple tools for FBA that can be picked up quickly by new users.

The metabolic networks that are used for FBA simulations are represented by genome-scale models (GEMs). GEMs are available for many model organisms, with the number growing rapidly [4]. Standard formats with clear specifications and workflows for creating high quality GEMs are available [5]. Any new tool for FBA needs to support import of the many existing models. Genome-scale models of key model organisms have many thousands of metabolites and reactions [6–8], so it can be very difficult for newcomers and experienced modelers to recognize the changes in FBA simulations. This is where visualizations of GEMs and associated data play a critical role in the modeling process [1].

The most popular software packages for FBA simulation require computer programming skills. COBRA Toolbox [9] and COBRApy [10] require knowledge of the MATLAB and Python programming languages, respectively. PSAMM is yet another Python-based toolbox [11]. The only popular application for FBA that does not require computer programming is OptFlux [12]. Therefore, OptFlux has become popular as a teaching tool for FBA and other strain design algorithms. OptFlux and COBRA Toolbox both have FBA visualization features, and there are dedicated visualization tools like FBASimVis [13] and FluxViz [14] available. FAME also provides a web-based environment for FBA simulation and visualization [15]. However, the best way to understand FBA is to explore simulations interactively by modifying parameters and receiving immediate feedback. Until now, no software has enabled this kind of interactive exploration of FBA simulations, and no existing FBA tools are fully web-based.

Escher-FBA meets these needs by extending the Escher application for pathway visualization with on-the-fly FBA calculations. Escher-FBA adds functionality so users can modify parameters in the FBA simulation—including flux bounds, objective function, and reaction knockouts—with immediate visualization of the results. Escher-FBA constructs the network and reaction data using the same input files as Escher. Additionally, since Escher-FBA is a web application, it works across platforms, including mobile devices. These features make Escher-FBA an ideal choice for academic labs and classrooms where students and researchers need a cross-platform visualization tool for learning and exploring FBA simulations.

## Implementation

Escher-FBA is built upon Escher [16], a versatile and user-friendly visualization tool for metabolic pathways. Users can quickly and easily create their own maps by first loading a GEM containing all the reactions in the system and then building visualizations comprised of both reactions (symbolized by arrows) and metabolites (symbolized by circles). Users can also load, modify, and save their maps, as well as maps that have been created by the community. Escher maps are stored as JavaScript Object Notation (JSON) files.

**Fig. 1:**
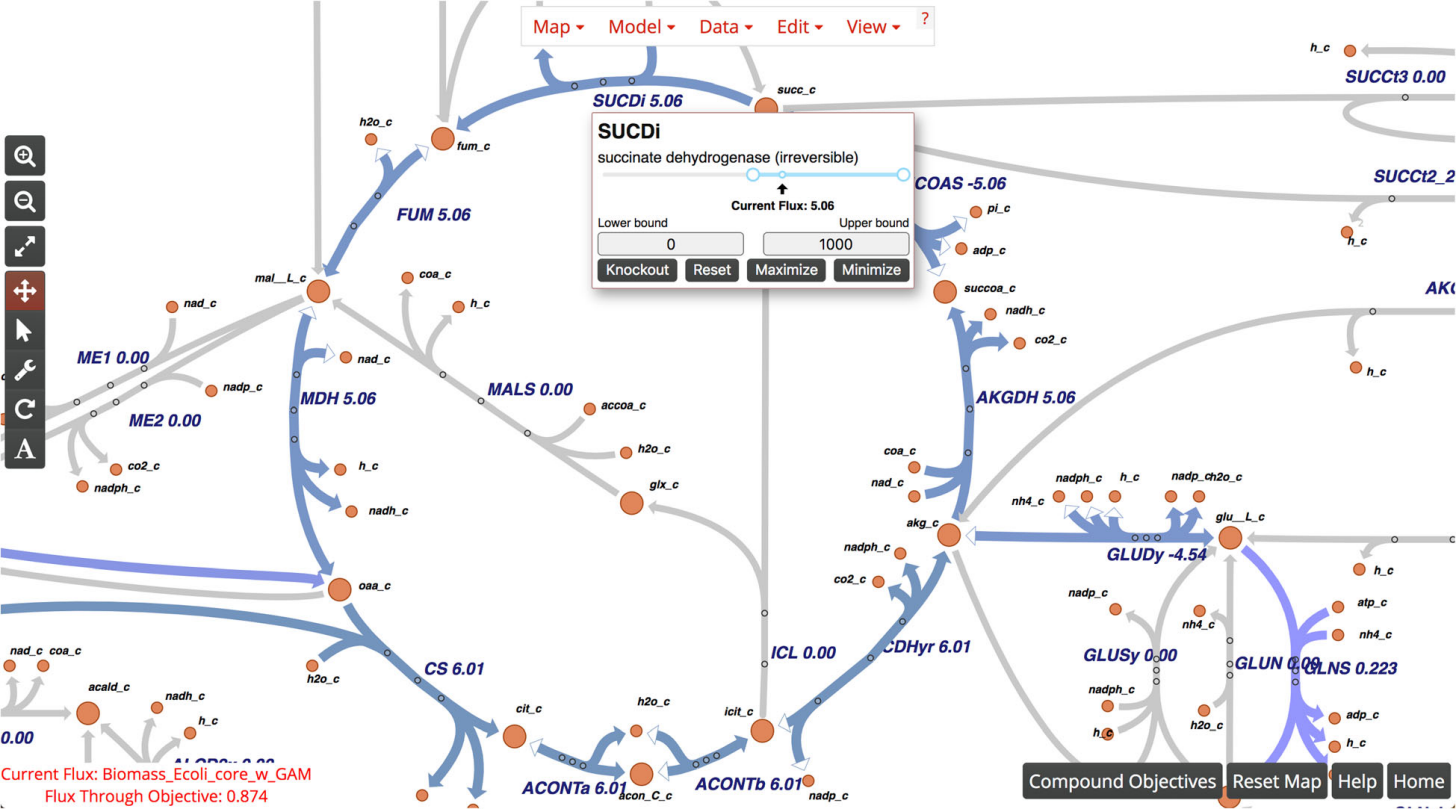
A screenshot of Escher-FBA. Buttons inside the tooltip for each reaction allow for quick modifications to be made to the network by the user with immediate visual updates to the network. At the bottom of the screen the current objective function, flux through the objective, and a reset button for the whole map can be seen

Escher-FBA extends Escher with interactive tooltips that appear when a user hovers the mouse over (or taps on a touchscreen) any reaction in the pathway visualization (Fig. 1). These tooltips contain controls that immediately modify the parameters of the FBA simulation. FBA simulations are executed using the GNU Linear Programming Kit (GLPK), which has been compiled to JavaScript and runs in the browser (https://github.com/hgourvest/glpk.js). The slider control in the tooltip adjusts both the upper and lower flux bounds of the reaction, while also displaying the current flux through the reaction. Value fields for upper and lower flux bounds allow the users to enter precise values. A **Knockout** button can be used to simulate a knockout of the associated reaction by setting both the upper and lower bounds of the reaction to zero. There is also a **Reset** button to reset the bounds to their original values from the loaded model. Finally, the **Maximize** and **Minimize** buttons can be used to change the FBA objective function to maximize or minimize the flux through the current reaction. The current objective and flux through that objective are displayed along the bottom-left corner of the screen, and buttons to reset the entire map back to its original data and to view a help menu are in the bottom-right corner of the screen. Perturbing the system with these controls produces immediately visible effects within the system. There is no need to manually re-run the simulation as Escher-FBA produces a new solution in response to user input.

Escher-FBA supports upload of custom maps and models, using the same upload functionality and file format as Escher [16]. Users can create their own maps and models and perform in silico experiments with their data. Additional maps and models can be downloaded from BiGG Models (http://bigg.ucsd.edu) [17]. The default map and model in Escher-FBA is a core model of central glucose metabolism in *E. coli* K-12 MG1655 (available at http://bigg.ucsd.edu/models/e_coli_core). This model is small enough that the user can see everything happening in the simulation within a single pathway map. Escher-FBA also supports full GEMs. Any GEM in the COBRA JSON file format can be imported in Escher-FBA directly. Models in other formats can be converted to JSON using COBRApy [10]. COBRApy supports many other file formats, including the latest Systems Biology Markup Language (SBML) with the Flux Balance Constraints (FBC) extension [18]. The JSON specification for COBRA models is also being adopted by other tools [11].

## Results and discussion

In order to demonstrate the use of Escher-FBA for real applications, we present four key FBA examples that can be executed directly in the browser. These are adapted from a review of FBA and its applications [2]. These examples rely on the default core model of *E. coli*, so they are ready to be implemented as soon as the Escher-FBA webpage is opened. Make sure to click the **Reset Map** button between each example. If you are having trouble finding a reaction, simply click the **Find** option in the **View** menu (or the “f” key on your keyboard) to open up a search bar.

### FBA with alternate carbon substrates

**Fig. 2:**
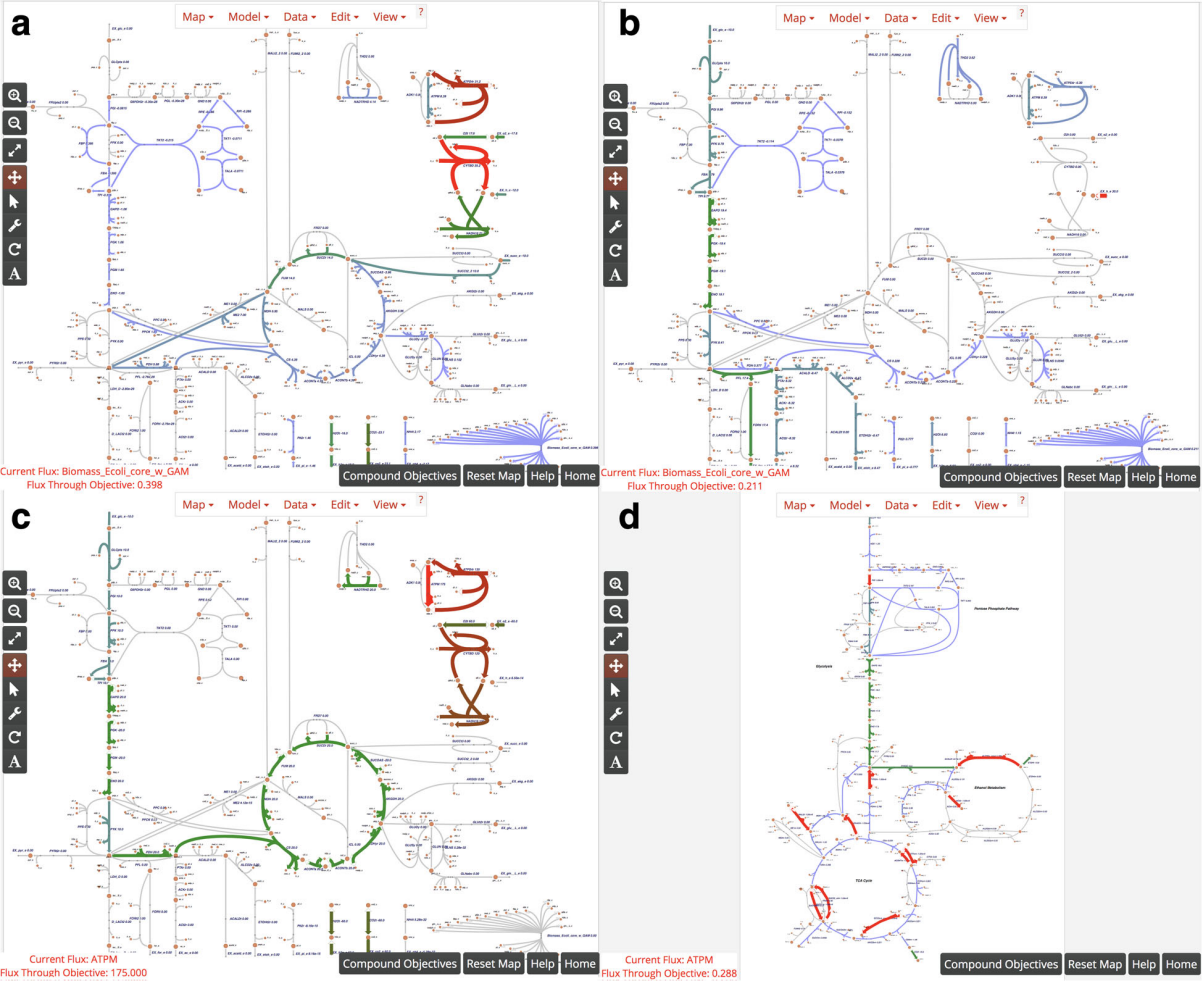
Examples of Escher-FBA simulations. (**a**) Simulated growth with succinate as sole carbon source. (**b**) Simulated anaerobic growth on a glucose minimal medium. (**c**) Maximizing ATP yield in the default model. (**d**) Growth of the *i*MM904 model of *S. cerevisiae*. Note that arrow widths were increased in the settings menu to make changes more obvious

The first example demonstrates the use of FBA to predict whether growth can occur on alternate carbon substrates. The default core model of *E. coli* includes a simulated minimal medium with D-glucose as the carbon source. Here, we will switch the carbon source from D-glucose to succinate. First, mouse over the succinate exchange reaction **EX_succ_e**, and change the lower bound to − 10 mmol/gDW/hr., either by dragging the slider or by entering − 10 into the **Lower Bound** field. Next, mouse over the D-glucose exchange reaction **EX_glc_e**, and either raise the lower bound to 0 or click the **Knockout** button. The default objective is still to maximize growth, so these two changes will instruct the program to calculate the maximum growth rate while using succinate as the carbon source instead of D-glucose. You should see that the maximum predicted growth rate decreases from 0.874 h^− 1^ to 0.398 h^− 1^, reflecting the lower growth yield of *E. coli* on succinate (Fig. 2a). This is the general approach to making changes in Escher-FBA; mouse over the reaction, make the required changes, and Escher-FBA will automatically display your results. The lower bound values for carbon source exchange represent experimental measurements, so you can try adjusting the specific lower bound value to realistic values for growth on other carbon sources.

### FBA during anaerobic growth

Anaerobic growth can be simulated in the same way by mousing over the **EX_o2_e** reaction and either clicking **Knockout** or changing the lower bound to 0. If you change oxygen exchange to zero while succinate is still the only carbon source, the **Flux Through Objective** indicator displays “Infeasible solution/Dead cell”, meaning that growth is not possible. Try clicking the **Reset** button in the bottom right corner to simulate a minimal medium with D-glucose as a carbon source, then knock out **EX_o2_e**, and the predicted growth rate should be 0.211 h^− 1^ (Fig. 2b).

### FBA with compound objectives

Escher-FBA supports setting of multiple objectives in the Compound Objectives mode. In the default model, setting a new objective always disables the previous objective. To enable the mode, first click the **Compound Objectives** button on the bottom of the screen. As an example of using the mode: to check the maximal growth rate while minimizing flux through **SUCDi**, start with the default objective of maximizing biomass production. Then, mouse over the reaction label for **SUCDi** and click the **Minimize** button within the tooltip. In the bottom right, you should see both objectives listed. Note that only objective coefficients of 1 or − 1 (represented by Maximize and Minimize) are currently supported. To go back to single objectives, just click the **Compound Objectives** button again.

### Analysis of metabolic yields

We can also use Escher-FBA to determine the maximum yields of precursors and cofactors such as ATP. All that is required is a stoichiometrically balanced reaction that consumes the cofactor of interest. The ATP Maintenance (**ATPM**) reaction is one such example. In order to determine the maximum production of ATP, simply mouse over the **ATPM** reaction and click the **Maximize** button. Setting up the objective this way works because, in order for the system to maximize flux through the **ATPM** reaction, it must first produce ATP in the highest possible quantity. When **ATPM** is maximized in the default core metabolism model of *E. coli*, the objective value is 175 mmol/gDW/hr. (Fig. 2c). With succinate as a carbon source, this value decreases to 82.5 mmol/gDW/hr. This same procedure can be followed for any metabolite of interest by creating a stoichiometrically-balanced consumption reaction and setting the model to maximize the flux through that reaction. Note that it is not currently possible to create such a reaction automatically in Escher-FBA, but this can be added to a future release.

### Flux variability analysis

Analyzing alternate optimal solutions in metabolism is another useful application of FBA [19]. Since the solutions produced through FBA are often non-unique, it can be useful to know the range of flux values a particular reaction can have. Flux variability analysis (FVA) is often used to calculate these ranges across the entire network [20]. Escher-FBA does not support FVA calculations directly, but it is possible to calculate them for a given reaction. In order to do this, first mouse over the objective function (the biomass reaction **Biomass_Ecoli_core_w_GAM**) and set the upper and lower bounds to slightly less than the current flux value (in the default map, try 0.870). Next, mouse over a reaction of interest and click the **Maximize** and **Minimize** buttons to see the maximum and minimum flux through that reaction given the optimal growth rate. For example, maximizing and minimizing flux through **GAPD** in glycolysis yields a feasible flux range of 15.44–16.68 mmol/gDW/hr., indicating that glycolytic flux is highly constrained at high growth rates. On the other hand, maximizing and minimizing flux through **MALS** in the glyoxylate shunt yields a feasible flux range of 0–2.64 mmol/gDW/hr., indicating that the glyoxylate shunt can be activate or inactive at high growth rates. This procedure can be done with any set of reactions and the user can constrain their system to any number of flux values to see the range of solutions available to a particular reaction.

### Using other genome-scale models

The default *E. coli* core model is not the only system that can be simulated. For example, if one wishes to run simulations on a yeast cell, a model and map for *Saccharomyces cerevisiae* can be downloaded from http://bigg.ucsd.edu/models/iMM904. On that page, click the download button for the model (iMM904.json) and the map (iMM904.Central carbon metabolism.json). Load these in Escher-FBA by clicking **Load Map JSON** in the **Map** menu and **Load Model JSON** in the **Model** menu to load both JSON files. Once loaded, the map is ready to edit and simulate with any of the tools in Escher or Escher-FBA (Fig. 2d). With a larger model like iMM904, not all reactions will be visible at once, but you can add a reaction to the visualization. First either click the wrench icon on the sidebar or select **Add reaction mode** from the **Edit** menu. Now, reactions can be added by clicking anywhere on the map and selecting the desired reaction from the drop down menu. The text input field can be used to search for a reaction of interest.

### Application of Escher-FBA to microbial cell factory design

To provide an example of a research hypothesis that can be tested using Escher-FBA, we loaded genome-scale models of *E. coli* containing two routes to produce 1-propanol for chemical production. These pathways were recently analyzed in a study on the predictive power of genome-scale models for simulating real microbial cell factory strains [21]. The first model includes a single route to 1-propanol production (Additional file 1) first reported by Atsumi et al. [22]. The second model includes two synergistic pathways for 1-propanol production (Additional file 2) first reported by Shen and Liao [23]. Each model can be loaded separately (with the **Model > Load COBRA model JSON** menu button), and a single map of central metabolism is provided that is compatible with both models (Additional file 3, can be loaded with **Map > Load Map JSON**).

**Fig. 3:**
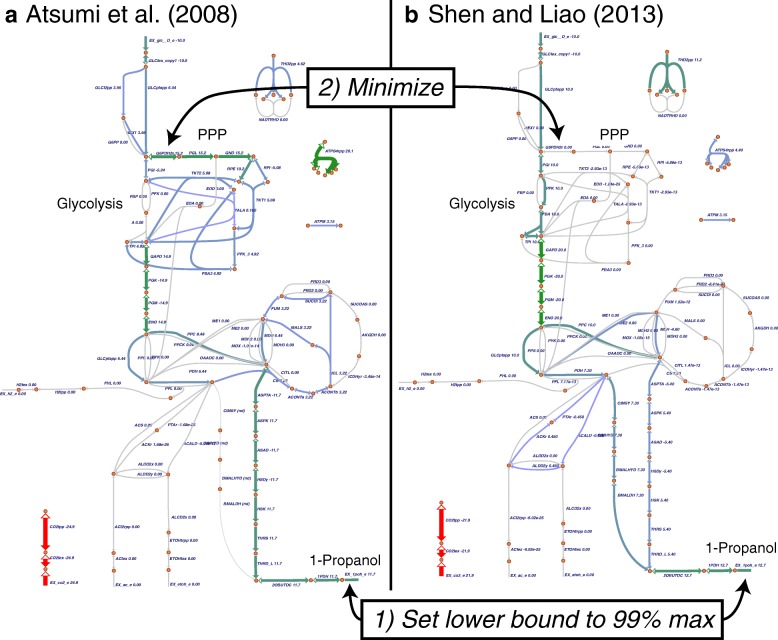
Pathway usage for two heterologous routes to 1-propanol production in *E. coli*. The pentose phosphate pathway (PPP) flux necessary for each heterologous production pathway can be compared by, first, forcing production of 1-propanol to be 99% of the maximum value (by setting the lower bound of the 1-propanol exchange reaction) and, second, minimizing flux through the first step in the PPP. (**a**) The 1-propanol pathway reported by Atsumi et al. [22] uses a single path to achieve 1-propanol production. It requires significant PPP flux and has a lower overall yield. (**b**) The pathway reported by Shen and Liao [23] uses two pathway synergistically to achieve higher yield. The pathway is stoichiometrically balanced with glycolysis, so it requires no PPP flux

We were curious whether the synergistic approach to 1-propanol production—which is known to have higher production yield—also has a difference in required pathway usage. Therefore, we loaded each model individually, maximized the excretion of 1-propanol (hovered over EX_1poh_e and clicked **Maximize**), set the lower bound for excretion to 99% of the maximum, then minimized the flux through the first committed step of the pentose phosphate pathway, glucose 6-phosphate dehydrogenase (G6PDH2r). The resulting maps demonstrate that the synergistic pathways for 1-propanol production are stoichiometrically balanced with glycolysis, so they do not require PPP activity (Fig. 3b). On the other hand, the individual pathway requires significant PPP flux (Fig. 3a). Other pathway usage, such as the necessary TCA flux for each case, can also be explored on these maps.

While Escher-FBA can already be used for many FBA simulations directly in the web browser, a number of the examples presented by Orth et al. cannot currently be accomplished with Escher-FBA [2]. As of right now, Escher-FBA cannot perform functions such as gene knockout analysis or robustness analysis. However, Escher-FBA uses flexible SVG representations for visual elements, so robustness analysis and even graphical features such as phase planes could be added. We have established a development roadmap for Escher-FBA (available from the homepage https://sbrg.github.io/escher-fba) and an iterative development process [24] to eventually enable complex systems biology analysis in the web browser.

## Conclusions

Escher-FBA can perform interactive FBA simulations without any software installation or knowledge of computer programming. Additionally, even though it does not perform the advanced analysis available in popular libraries like the COBRA Toolbox and COBRApy, Escher-FBA can serve as an entry point for new users of FBA, for teaching FBA to students, and as a research tool for experts who want to visually explore simulations.

## Availability and requirements

**Project name:** Escher-FBA.

**Project home page:** https://sbrg.github.io/escher-fba

**Operating system:** Platform independent.

**Programming language:** JavaScript.

**Any restrictions to use by non-academics:** none.

## Additional files

Additional file 1: **iJO1366 Atsumi2008.json.** The genome-scale model of *E. coli* that contains the 1-propanol production pathway reported by Atsumi et al. [22]. (JSON 1224 kb)

Additional file 2: **iJO1366 Shen2013.json.** The genome-scale model of *E. coli* that contains the 1-propanol production pathway reported by Shen and Liao [23]. (JSON 1224 kb)

Additional file 3: **iJO1366.Central metabolism 1POH.json.** The Escher map that is compatible with both models (Additional files 1 and 2). (JSON 168 kb)

## Abbreviations

FBA: Flux Balance Analysis
FBC: Flux Balance Constraints
FVA: Flux Variability Analysis
GEM: Genome-scale Model
GLPK: GNU Linear Programming Kit
JSON: JavaScript Object Notation
SBML: Systems Biology Markup Language

## References

[1] Bordbar Aarash, Monk Jonathan M., King Zachary A., Palsson Bernhard O. (2014). Constraint-based models predict metabolic and associated cellular functions. Nature Reviews Genetics.

[2] Orth JD, Thiele I, Palsson BØ (2010). What is flux balance analysis?. Nat Biotechnol [Internet]..

[3] Lewis Nathan E., Nagarajan Harish, Palsson Bernhard O. (2012). Constraining the metabolic genotype–phenotype relationship using a phylogeny of in silico methods. Nature Reviews Microbiology.

[4] Monk J, Nogales J, Palsson BO (2014). Optimizing genome-scale network reconstructions. Nat Biotechnol [Internet]. Nature Publishing Group.

[5] Thiele I, Palsson BØ (2010). A protocol for generating a high-quality genome-scale metabolic reconstruction. Nat Protoc [Internet]. Nature Publishing Group.

[6] Brunk Elizabeth, Sahoo Swagatika, Zielinski Daniel C, Altunkaya Ali, Dräger Andreas, Mih Nathan, Gatto Francesco, Nilsson Avlant, Preciat Gonzalez German Andres, Aurich Maike Kathrin, Prlić Andreas, Sastry Anand, Danielsdottir Anna D, Heinken Almut, Noronha Alberto, Rose Peter W, Burley Stephen K, Fleming Ronan M T, Nielsen Jens, Thiele Ines, Palsson Bernhard O (2018). Recon3D enables a three-dimensional view of gene variation in human metabolism. Nature Biotechnology.

[7] Monk Jonathan M, Lloyd Colton J, Brunk Elizabeth, Mih Nathan, Sastry Anand, King Zachary, Takeuchi Rikiya, Nomura Wataru, Zhang Zhen, Mori Hirotada, Feist Adam M, Palsson Bernhard O (2017). iML1515, a knowledgebase that computes Escherichia coli traits. Nature Biotechnology.

[8] Aung HW, Henry SA, Walker LP (2013). Revising the Representation of Fatty Acid, Glycerolipid, and Glycerophospholipid Metabolism in the Consensus Model of Yeast Metabolism. Ind Biotechnol [Internet]..

[9] Schellenberger Jan, Que Richard, Fleming Ronan M T, Thiele Ines, Orth Jeffrey D, Feist Adam M, Zielinski Daniel C, Bordbar Aarash, Lewis Nathan E, Rahmanian Sorena, Kang Joseph, Hyduke Daniel R, Palsson Bernhard Ø (2011). Quantitative prediction of cellular metabolism with constraint-based models: the COBRA Toolbox v2.0. Nature Protocols.

[10] Ebrahim Ali, Lerman Joshua A, Palsson Bernhard O, Hyduke Daniel R (2013). COBRApy: COnstraints-Based Reconstruction and Analysis for Python. BMC Systems Biology.

[11] Dufault-Thompson Keith, Steffensen Jon Lund, Zhang Ying (2017). Using PSAMM for the Curation and Analysis of Genome-Scale Metabolic Models. Methods in Molecular Biology.

[12] Rocha Isabel, Maia Paulo, Evangelista Pedro, Vilaça Paulo, Soares Simão, Pinto José P, Nielsen Jens, Patil Kiran R, Ferreira Eugénio C, Rocha Miguel (2010). OptFlux: an open-source software platform for in silico metabolic engineering. BMC Systems Biology.

[13] Grafahrend-Belau E., Klukas C., Junker B. H., Schreiber F. (2009). FBA-SimVis: interactive visualization of constraint-based metabolic models. Bioinformatics.

[14] König M, Holzhütter H-G. Fluxviz - Cytoscape plug-in for visualization of flux distributions in networks. Genome Inform [Internet], Available from. 2010;24:96–103 https://www.ncbi.nlm.nih.gov/pubmed/22081592. 22081592

[15] Boele Joost, Olivier Brett G, Teusink Bas (2012). FAME, the Flux Analysis and Modeling Environment. BMC Systems Biology.

[16] King Zachary A., Dräger Andreas, Ebrahim Ali, Sonnenschein Nikolaus, Lewis Nathan E., Palsson Bernhard O. (2015). Escher: A Web Application for Building, Sharing, and Embedding Data-Rich Visualizations of Biological Pathways. PLOS Computational Biology.

[17] King Zachary A., Lu Justin, Dräger Andreas, Miller Philip, Federowicz Stephen, Lerman Joshua A., Ebrahim Ali, Palsson Bernhard O., Lewis Nathan E. (2015). BiGG Models: A platform for integrating, standardizing and sharing genome-scale models. Nucleic Acids Research.

[18] Olivier BG, Bergmann FT. The Systems Biology Markup Language (SBML) Level 3 Package: Flux Balance Constraints. J Integr Bioinform [Internet]. 2015;12:269. Available from: 10.2390/biecoll-jib-2015-269 10.2390/biecoll-jib-2015-269 26528567

[19] Feist Adam M., Zielinski Daniel C., Orth Jeffrey D., Schellenberger Jan, Herrgard Markus J., Palsson Bernhard Ø. (2010). Model-driven evaluation of the production potential for growth-coupled products of Escherichia coli. Metabolic Engineering.

[20] Mahadevan R., Schilling C.H. (2003). The effects of alternate optimal solutions in constraint-based genome-scale metabolic models. Metabolic Engineering.

[21] King ZA, O’Brien EJ, Feist AM, Palsson BO (2017). Literature mining supports a next-generation modeling approach to predict cellular byproduct secretion. Metab Eng [Internet]. Cold Spring Harbor Labs Journals.

[22] Atsumi Shota, Hanai Taizo, Liao James C. (2008). Non-fermentative pathways for synthesis of branched-chain higher alcohols as biofuels. Nature.

[23] Shen Claire R., Liao James C. (2013). Synergy as design principle for metabolic engineering of 1-propanol production in Escherichia coli. Metabolic Engineering.

[24] Yurkovich James T., Yurkovich Benjamin J., Dräger Andreas, Palsson Bernhard O., King Zachary A. (2017). A Padawan Programmer’s Guide to Developing Software Libraries. Cell Systems.

